# FNDC5 Causes Resistance to Sorafenib by Activating the PI3K/Akt/Nrf2 Pathway in Hepatocellular Carcinoma Cells

**DOI:** 10.3389/fonc.2022.852095

**Published:** 2022-03-22

**Authors:** Huayuan Liu, Lei Zhao, Mengya Wang, Kexin Yang, Zhipeng Jin, Chengjian Zhao, Guangjun Shi

**Affiliations:** ^1^ Department of Hepatobiliary Surgery, The Affiliated Qingdao Municipal Hospital of Qingdao University, Qingdao, China; ^2^ Department of Infection Management, The Affiliated Qingdao Municipal Hospital of Qingdao University, Qingdao, China; ^3^ Department of Physiology, School of Basic Medicine, Institute of Brain Science and Disorders, Qingdao University, Qingdao, China; ^4^ Department of Gynecology, The Affiliated Qingdao Municipal Hospital of Qingdao University, Qingdao, China

**Keywords:** fibronectin type III domain containing 5, ferroptosis, hepatocellular carcinoma, PI3K/Akt/Nrf2 pathway, sorafenib

## Abstract

In this study, we aimed to reveal the resistance mechanism of hepatocellular carcinoma (HCC) cells to sorafenib by exploring the effect of FNDC5 on sorafenib-induced ferroptosis in HCC cells. We compared the expression level of FNDC5 between sorafenib-resistant and sorafenib-sensitive HCC cell lines and the level of ferroptosis between the groups after treatment with sorafenib. We knocked down FNDC5 in drug-resistant cell lines and overexpressed it in sorafenib-sensitive HCC cell lines to further demonstrate the role of FNDC5 in sorafenib-induced ferroptosis. Using PI3K inhibitors, we revealed the specific mechanism by which FNDC5 functions. In addition, we verified our findings obtained in *in vitro* experiments using a subcutaneous tumorigenic nude mouse model. The findings revealed that FNDC5 inhibits sorafenib-induced ferroptosis in HCC cells. In addition, FNDC5 activated the PI3K/Akt pathway, which in turn promoted the nuclear translocation of Nrf2 and increased the intracellular antioxidant response, thereby conferring resistance to ferroptosis. Our study provides novel insights for improving the efficacy of sorafenib.

## Introduction

Hepatocellular carcinoma (HCC) has become a serious threat to human health because of its high malignancy and occurrence worldwide (1). Despite recent advances in the exploration of treatments and mechanisms of occurrence, the overall prognosis of HCC remains poor owing to factors such as insidious onset and susceptibility to drug resistance (2).

Currently, sorafenib is used as a first-line agent for the treatment of advanced HCC; it mainly acts through the modulation of the RAF/MEK/ERK pathway and vascular endothelial growth factor receptor (VEGF), thereby inhibiting tumor growth (3). Recent studies have shown that sorafenib-treated HCC cells undergo specific ferroptosis-related manifestations, such as lipid peroxidation, glutathione depletion, and iron accumulation (4–6). These events lead to HCC cell death; thus, induction of ferroptosis could improve the anticancer effect of sorafenib and might be a new therapeutic strategy for HCC (7).

Nuclear factor E2-related factor 2 (Nrf2), which mainly regulates the transcription of intracellular antioxidant enzymes, can improve the antioxidant capacity of cancer cells, thereby inhibiting the occurrence of ferroptosis in HCC cells (8). Moreover, it is a key factor in the resistance of cancer cells to sorafenib. Studies have found that Nrf2 is activated by the PI3K/Akt pathway (9, 10), and abnormal activation of the PI3K/Akt pathway is also an important factor leading to resistance to sorafenib in HCC (11, 12).

Fibronectin type III domain containing 5 (FNDC5) is a recently identified factor associated with electron transport in mitochondrial oxidative respiration, which converts white adipose tissue to brown adipose tissue (13, 14). Several inflammation-related studies have revealed that FNDC5 might affect the activation of Nrf2 (15–17). Furthermore, in a previous study, we found that FNDC5 activates the PI3K/Akt pathway in HCC cells (18). However, the specific mechanism of FNDC5 in hepatocarcinogenesis needs further exploration. In the present study, we aimed to reveal the antioxidant role of FNDC5 in the treatment of HCC with sorafenib. We found that FNDC5 might activate Nrf2 through the PI3K/Akt pathway, leading to the development of ferroptosis resistance in HCC cells, and thus resistance to sorafenib.

## Materials and Methods

### Ethical Statement

Tumor tissue specimens from patients were obtained in strict accordance with the regulations of the research ethics committee of Qingdao Municipal Hospital. In addition, all immunohistochemistry experiments were performed under the supervision of personnel of the research ethics committee. Animal experiment protocols were designed in strict accordance with the ARRIVE guidelines and reviewed and approved by the research ethics committee of Qingdao Municipal Hospital. All experimental procedures were recorded, reviewed, and completed under the supervision and guidance of the research ethics committee (ethics approval number: 104).

### Immunohistochemistry

Immunohistochemistry was performed as previously described (19). Tumor and adjacent noncancerous (normal) liver tissues (60 pairs in total) were obtained from patients with liver cancer, fixed with 4.0% paraformaldehyde, paraffin-embedded, and cut into 4-μm-thick sections. The sections were treated with 3.0% hydrogen peroxide and blocked with 5.0% bovine serum, and then incubated with FNDC5 (1:200, ab181884; Abcam, Cambridge, MA, USA) and Nrf2 (1:200, 16396-1-AP, Proteintech, Beijing, China) antibodies overnight at 4°C. After washing with PBS, the sections were incubated with secondary antibodies conjugated with species-specific horseradish peroxidase (HRP) for 1 h at 25°C, and finally stained with diaminobenzidine (DAB) and hematoxylin. Images were taken using an AxioVision Rel.4.6 computerized image analysis system (Carl Zeiss). The degree of staining was assessed based on the staining area and intensity, using to the following formula: degree of staining = area × intensity. Staining area was scored as follows: 0 (staining area close to 0%), 1 (staining area less than 10%), 2 (staining area between 10% and 35%), 3 (staining area between 35% and 75%), and 4 (staining area between 76% and 100%). Staining intensity was scored as follows: 0 (no staining), 1 (weak staining), 3 (moderate staining), and 4 (strong staining). The final expression level of the target protein is expressed as the SI score, which was calculated using the following formula: SI = staining area score × staining intensity score, with SI ≥ 8 indicating high expression, whereas SI < 8 indicating low expression.

### Cell Culture

HepG-2 and Huh7 human HCC cell lines were provided by the Chinese Academy of Sciences Cell Bank (Shanghai, China). The cells were cultured in Dulbecco’s modified Eagle’s medium (DMEM; HyClone, USA) containing 10% fetal bovine serum (FBS; Excellbio, USA) and 1% penicillin–streptomycin (HyClone, USA). All cells were incubated at 37°C under 5% CO_2_.

### Culture of Drug-Resistant Cell Lines

The HCC cells were incubated in 96-well plates with different concentrations of sorafenib, and the half-inhibitory concentration (IC_50_) was determined. The HCC cells were then cultured in six-well plates at a density of 10^4^ cells per well and treated with sorafenib at concentrations slightly below the IC_50_ value. The concentration of sorafenib was increased by 0.25 µmol/L every week until the maximum tolerated concentration of 10 µM was reached; at this point, the HepG2-SR- and Huh7-SR-resistant cell lines were obtained and cultured continuously in a medium with sorafenib at 1 µM to ensure the persistence of drug resistance.

### Cell Viability Assay

The HCC cells were inoculated in 96-well plates at a density of 5 × 10^3^ cells/well and incubated for 12 h, and then treatment with different concentrations (1, 2, 4, 8, 16, 32, 64, and 128 µM) of sorafenib for 24 h. The medium was replaced with fresh medium; there were six replicates per group. The Cell Counting Kit-8 (CCK8) reagent (APExBIO, K1018, USA) was added into the wells according to the manufacturer’s instructions. After incubating the plates for 1 h in the dark, the absorbance of the samples was measured at 450 nm using a microplate reader (Bio-Tek, VT, USA). Cell survival was calculated using the following formula: Cell survival (%) = (test group OD − negative control value)/(control group OD − negative control OD) × 100. The IC_50_ was calculated using GraphPad Prism 7.0 software.

### Detection of Apoptosis Using Annexin-V-FITC/PI Staining

The cells cultured in six-well plates were collected, washed twice with PBS, and resuspended using the binding buffer available in the Annexin-V-FITC/PI kit; then, 5 μL of Annexin V-FITC and 5 μL of PI were successively added. The cells were stained in the dark for 15 min, and then immediately analyzed using flow cytometry.

### EdU Assay

Cell proliferation was determined using the BeyoClick EdU-594 kit (Beyotime, Shanghai, China). The cultured HCC cells were washed with PBS, and then 10 µM EdU and fresh medium were added; the cells were incubated at 37°C for 2 h. Subsequently, the cells were washed twice with PBS, fixed with 4% paraformaldehyde for 15 min, washed with PBS, and finally stained with DAPI for 5 min before washing with PBS. Images were acquired under a fluorescence microscope (IX70; Olympus, Tokyo, Japan).

### Knockdown and Overexpression of FNDC5

Human *FNDC5* cDNA was constructed by Gene Chem (Shanghai, China), packaged in a lentiviral plasmid vector, and used for cell transfection. The experimental group overexpressing *FNDC5* and negative control were named FNDC5-OE and FNDC5-NC2, respectively. siRNAs for knocking down *FNDC5* were constructed by RIBOBIO with the following sequences (5’-3’): 1. GGAGGATACGGAGTACATA; 2. AGAAGATGGCCTCCAAGAA; 3. GCTTCATCCAGGAGGTGAA. The first and second siRNAs exhibiting better knockdown effects were selected, and plasmids were constructed and packaged into lentiviral plasmid vectors by Gene Chem, using the component sequence hU6-MCS-CBh-gcGFP-IRES-puromycin. The experimental groups with *FNDC5* knockdown and negative control were named FNDC5-KD1, FNDC5-KD2, and FNDC5-NC1, respectively.

Following the culture of HepG-2 and Huh7 HCC cells and drug-resistant cell lines in six-well plates to reach 40% fusion, 10 µL of viral solution and 40 µL of infection enhancing solution were added, and the cells were transfected for 24 h. The complete medium was then replaced, and the culture was continued for 24 h. Finally, successfully transfected cells were screened using 1 µg/mL puromycin and used in the subsequent experiments.

### Transmission Electron Microscopy

The HCC cells were fixed with 2.5% glutaraldehyde for 1 h, washed three times with PBS, and fixed in 2% sodium tetroxide for 1 h. Thereafter, ethanol dehydration was performed, followed by sectioning and staining after embedding of the samples. Finally, the sections were observed using a Hitachi-7500 transmission electron microscope (Hitachi, Tokyo, Japan).

### Mitochondrial Membrane Potential Assay

JC-1 is a fluorescent probe that detects the ΔΨm mitochondrial membrane potential. When mitochondrial membrane potential decreases, JC-1 is converted from red fluorescent polymers (J-aggregates) to green fluorescent monomers (J-monomers). The HCC cells were incubated with JC-1 (Beyotime, Shanghai, China) in the dark at 37°C for 20 min, and then washed twice with staining buffer before analysis using a flow cytometer (Accuri C6, BD Biosciences, USA).

### Reactive Oxygen Species and Malondialdehyde Assays

The levels of reactive oxygen species (ROS) in HCC cells cultured in six-well plates were measured using the oxidation-sensitive fluorescent probe DCFH-DA (#D6883; Sigma) and analyzed using flow cytometry. After cell lysis, the concentration of malondialdehyde (MDA) was determined using the lipid peroxidation malondialdehyde assay kit (S0131S; Beyotime), according to the manufacturer’s instructions.

### Western Blotting

Total protein was extracted from HCC cells using the RIPA lysis buffer (Beyotime). Nuclei and cytoplasmic proteins were extracted using the nuclei and cytoplasmic protein extraction kit (Beyotime). Protein concentration was determined using a BCA protein quantification kit (Beyotime). The samples were then boiled for 10 min, followed by electrophoresis on 10% SDS-polyacrylamide gels, and the separated proteins were transferred on to polyvinylidene fluoride (PVDF) membranes (Millipore, Bedford, MA, USA). The membranes were then blocked with 5% skimmed milk and incubated at 25°C for 2 h. The membranes were then incubated with primary antibodies (1:1000, Proteintech) at 4°C overnight. Antibodies used included SLC7A11 (26864-1-AP), GPX4 (67763-1-Ig), DMT1 (20507-1-AP), PI3K (20584-1-AP), Akt (60203-2-Ig), p-Akt (66535-1-Ig), and Nrf2 (16396-1-AP), whereas β-actin (1:5000, #4967, Cell Signaling Technology, USA), and lamin B1 (1:2000, 12987-1-AP, Proteintech) were used as the internal references. The membranes were then washed thrice with TBST buffer and incubated with diluted HRP-coupled secondary antibodies for 2 h under 25°C. Finally, protein blots were detected using an ECL reagent (Millipore). Three independent replicate experiments were performed, and the intensity of protein blots was analyzed using ImageJ software.

### Xenograft Mouse Model

NOD-SCID (NOD CB17-Prkdcscid/NcrCrl) 5-week-old male mice were purchased from Beijing Life River Laboratory Animal Technology Company (Beijing, China). The mice were housed in a 12-h light/dark cycling environment. The temperature was 25°C ± 1°C, humidity was maintained at 56%, and the mice were provided adequate food and water. Mice weighing between 20 and 23 g were selected and 5 × 10^6^ Hep-G2 cells were subcutaneously injected into their backs. The mice were subsequently divided into the following four groups: control (n = 5), FNDC5 overexpressing (n = 5), FNDC5 overexpressing followed by treatment with the PI3K inhibitor LY294002 (MCE, China), and FNDC5 knockdown (n = 5). Seven days after cell injection, sorafenib (30 mg/kg) was administered to all mice *via* intraperitoneal injection every alternate day for 4 weeks. The mice in the third group were intraperitoneally injected with LY294002 (25 mg/kg) diluted with DMSO twice a week for 4 weeks. Tumor volumes were measured every 4 days, and the mice were sacrificed on day 28 using the cervical dislocation method. Tumor tissues were dissected out and processed into homogenates, lysed, and centrifuged, and the expression level of the target proteins was detected using western blotting. The levels of MDA in tumor tissues were detected using the MDA kit.

### Data Analysis

GraphPad 7 (GraphPad Software Inc., CA, USA) was used for data analysis. All experimental results represent data from at least three independent replicate experiments, and the results are expressed as mean ± SD. A *t*-test was used to compare the differences between groups. A one-way ANOVA was used to compare differences among multiple experimental groups. Results with p value < 0.05 were considered statistically significant.

## Results

### Expression of FNDC5 Was Elevated in Sorafenib-Resistant HCC Cells

**Figure 1:**
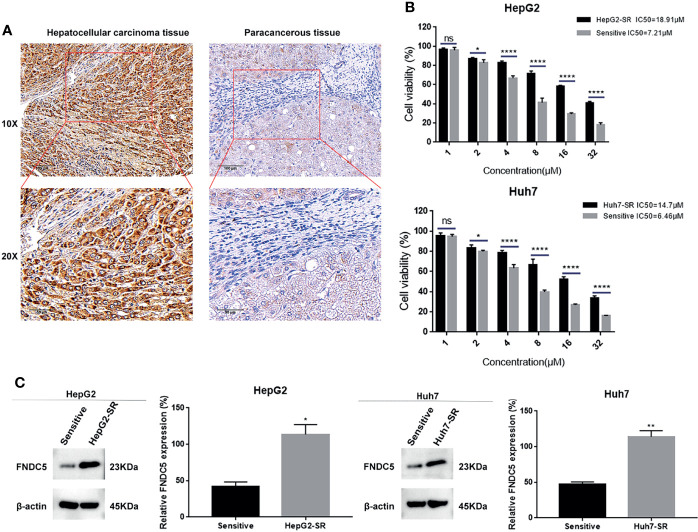
Elevated levels of FNDC5 in sorafenib-resistant HCC cells. **(A)** FNDC5 is overexpressed in HCC tissues. **(B)** IC_50_ values of sorafenib-sensitive and -resistant HCC cells were calculated using the CCK8 method after treating HCC cells with increasing concentrations of sorafenib for 24 h, and cell survival rates were measured. **(C)** Western blotting to detect differences in the expression of FNDC5 between sorafenib-sensitive and -resistant HCC cells. *Compared with the sorafenib-sensitive HCC cells, *p < 0.05, **p < 0.01, ****p < 0.0001. ns, no significant difference.

In our previous study, we found that FNDC5 was highly expressed in HCC tissues, promoting cancer progression (18). Our present immunohistochemical results also showed that among the 60 pairs of HCC and paraneoplastic tissues, the expression of FNDC5 was high in 48 HCC tissues, whereas its expression was low in 12 HCC and all paracancerous tissues (**Figure 1A**). To investigate whether the level of expression of FNDC5 was altered in sorafenib-resistant HCC cells, we treated HCC cell lines with increasing concentrations of sorafenib for 24 h. Following the CCK8 assay, we calculated the 24 h half-inhibitory concentration (IC_50_) of sorafenib in HepG2 and Huh7 cell lines, and by gradually increasing the concentration of sorafenib, we established sorafenib-resistant cell lines. We also examined the IC_50_ of the obtained resistant cell lines. We found that the IC_50_ of sorafenib for HepG2-SR and Huh7-SR cells was 2–3 times higher than that for sorafenib-sensitive HCC cells (**Figure 1B**). Following treatment of HCC cells with different concentrations of sorafenib for 24 h, we found that the viability of drug-resistant cells was significantly higher than that of the sorafenib-sensitive HCC cells (**Figure 1B**). After confirming the successful development of sorafenib-resistant HCC cells, we examined the expression of FNDC5 in HCC sorafenib-resistant and sorafenib-sensitive HCC cells using western blotting. We found that the expression of FNDC5 was significantly higher in resistant cells than in sorafenib-sensitive HCC cells (**Figure 1C**).

### Changes in the Expression of FNDC5 Caused Alterations in the Levels of Sorafenib-Induced Ferroptosis

**Figure 2:**
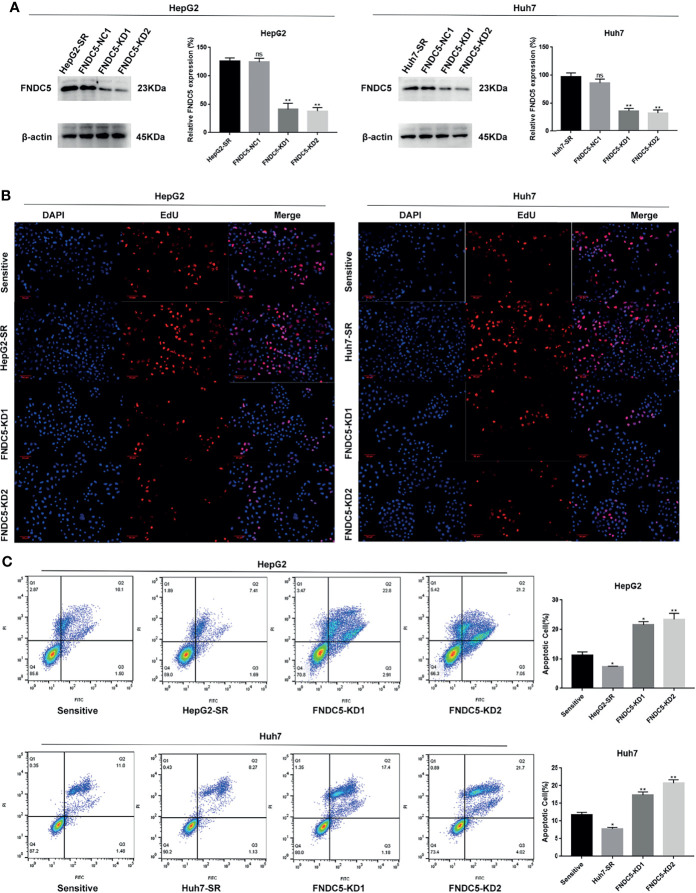
Knockdown of FNDC5 decreases drug resistance in sorafenib-resistant HCC cells. **(A)** A lentiviral plasmid vector was used to knockdown FNDC5 in drug-resistant cell lines; the knockdown effect was verified using western blotting. *Compared with the drug-resistant cell lines, *p < 0.05, **p < 0.01. Sorafenib (10 µM) was administered to sorafenib-sensitive HCC, drug-resistant, and FNDC5-knockdown drug-resistant cell lines for 24 h. **(B)** EdU staining was used to detect viability in each group of cells. **(C)** Annexin-V-FITC/PI staining was used to determine the inhibitory ability of sorafenib in each group of cells. *Compared with the sorafenib-sensitive HCC cells, *p < 0.05, **p < 0.01. ns, no significant difference.

**Figure 3:**
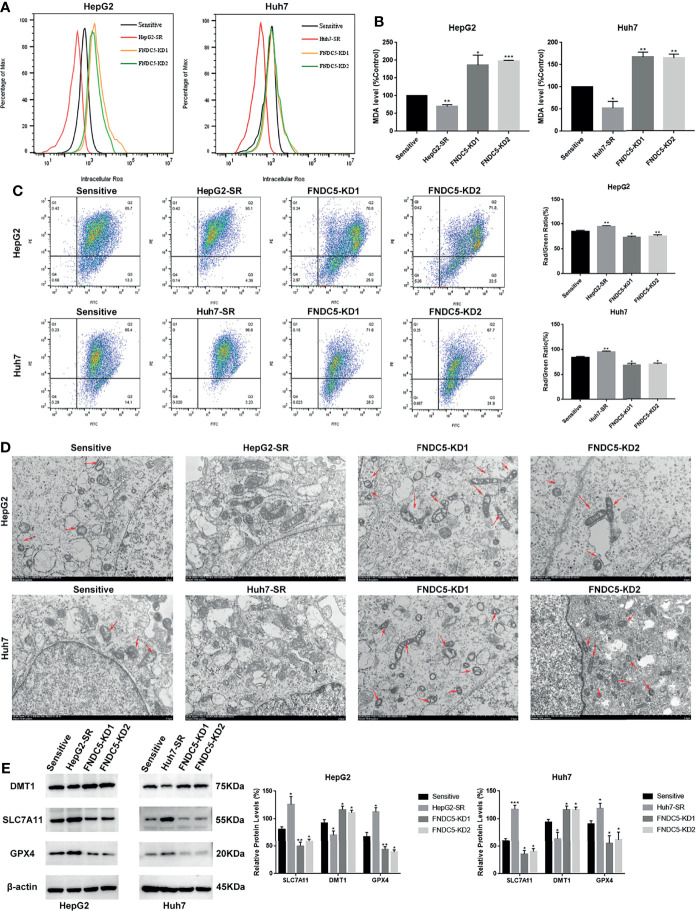
Effect of alterations in the expression level of FNDC5 on sorafenib-induced levels of ferroptosis in HCC cells. **(A, B)**. After 24 h of treatment of sorafenib-sensitive HCC cells, drug-resistant, and FNDC5-knockdown drug-resistant cells with sorafenib (10 µM), the levels of ROS and MDA were measured to assess the level of ferroptosis in each group of cells. **(C)**. Flow cytometry was used to detect changes in mitochondrial membrane potential in each group of cells. **(D)** The mitochondrial morphology in each group of cells was observed under an electron microscope, with red arrows indicating mitochondria with altered membrane potential. **(E)** Western blotting for detecting the level of ferroptosis markers in each group of cells. *Compared with the sorafenib-sensitive HCC cells, *p < 0.05, **p < 0.01, ***p < 0.001.

To further investigate whether the resistance of HepG2-SR and Huh7-SR cells to sorafenib was caused by changes in the expression of FNDC5, we knocked down *FNDC5* in HCC resistant cells (**Figure 2A**). The cells were then treated sorafenib-sensitive, drug-resistant, and *FNDC5*-knockdown drug-resistant cell lines using the same concentration of sorafenib, followed by staining with EdU and Annexin-V-FITC/PI to detect the inhibitory effect of sorafenib on each group (**Figures 2B** **, C**). We observed a higher rate of apoptosis in sorafenib-sensitive HCC cells, whereas drug-resistant HCC cells were obviously insensitive to sorafenib. However, we noticed that resistance to sorafenib was reversed after knocking down *FNDC5* in drug-resistant HCC cells. We then examined the alterations in the level of ferroptosis in each group of HCC cells through the evaluation of the levels of ROS and MDA (**Figures 3A** **, B**), JC-1 detection of mitochondrial membrane potential (**Figure 3C**), electron microscopic observation of mitochondria (**Figure 3D**), and detection of the changes in ferroptosis markers in each subgroup using western blotting (**Figure 3E**). We found that the sorafenib-induced level of ferroptosis was lower in sorafenib-resistant cells with high expression of FNDC5 than in sorafenib-sensitive HCC cells, whereas the level of ferroptosis was elevated in *FNDC5*-knockdown sorafenib-resistant cells.

### FNDC5 Inhibited Sorafenib-Induced Ferroptosis in Hepatocellular Carcinoma Cells Through the PI3K/Akt/Nrf2 Pathway

**Figure 4:**
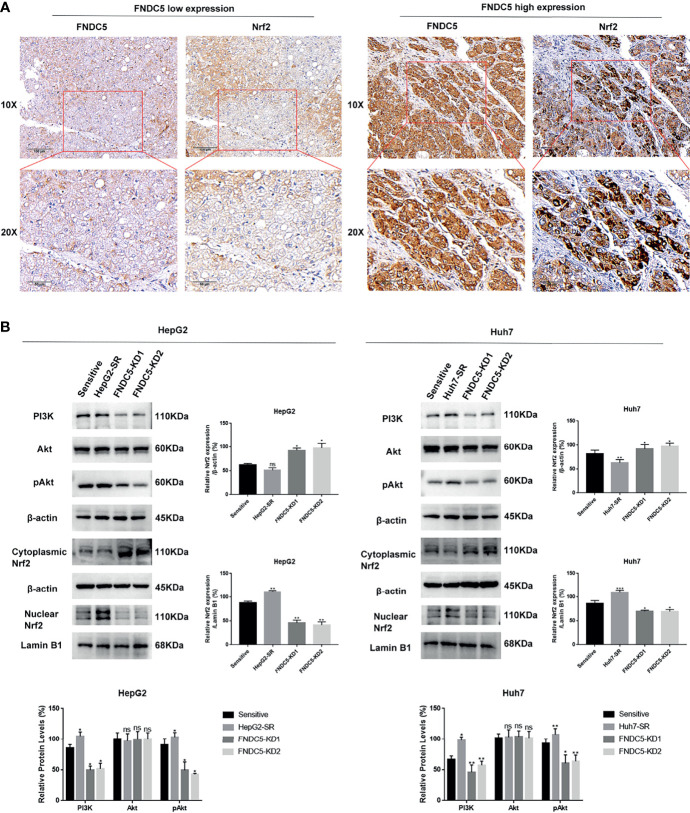
Changes in the expression level of FNDC5 affect the expression level of Nrf2. **(A)** Nrf2 expression was elevated in hepatocellular carcinoma tissues with high FNDC5 expression. **(B)** sorafenib-sensitive HCC cells, drug-resistant, and FNDC5-knockdown resistant cells were treated with sorafenib (10 µM) for 24 h, and the levels of PI3K/Akt/Nrf2 pathway proteins were detected in each group of cells using western blotting. *Compared with the sorafenib-sensitive HCC cells, *p < 0.05, **p < 0.01, ***p < 0.001. ns, no significant difference.

**Figure 5:**
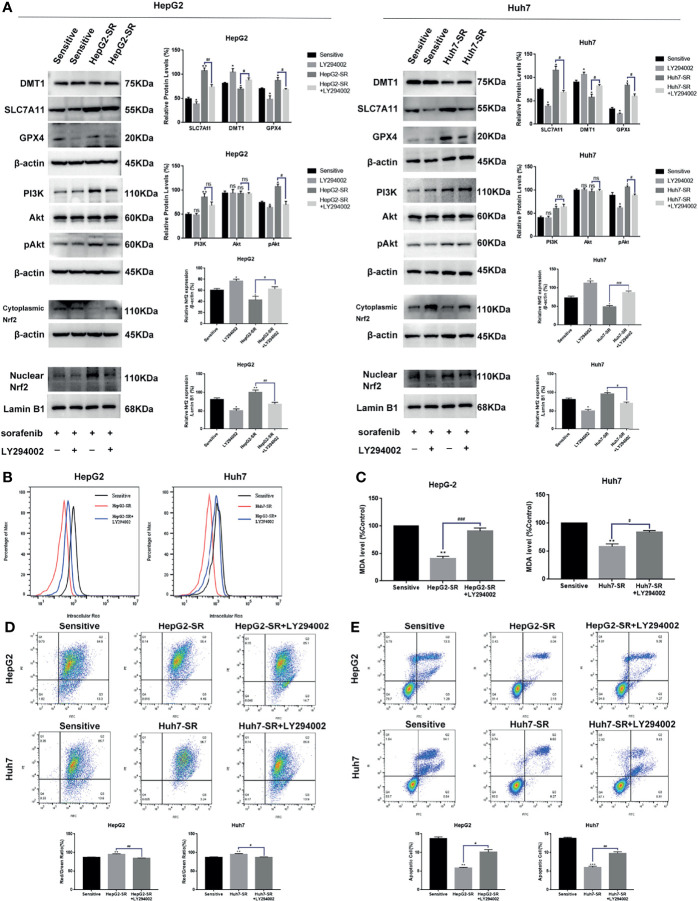
FNDC5 inhibits sorafenib-induced ferroptosis in HCC cells by activating the PI3K/Akt/Nrf2 pathway. Sorafenib (10 µM) was added to the medium of LY294002 (5 µM)-treated resistant, untreated resistant, and sorafenib-sensitive HCC cells and incubated for 24 h. **(A)** Western blotting of ferroptosis markers and PI3K/Akt/Nrf2 pathway proteins in each group of cells. **(B, C)**. Detection of levels of ROS and MDA. **(D)**. JC-1 staining for detecting mitochondrial membrane potential in each group of cells. **(E)**. Annexin-V-FITC/PI staining for detecting apoptosis rate in each group of cells. *Compared with the sorafenib-sensitive HCC cells, *p < 0.05, **p < 0.01, ***p < 0.001. ^#^Compared with HepG2-SR+LY294002 or Huh7-SR+LY294002, #p < 0.05, ^##^p < 0.01, ^###^p < 0.001. ns, no significant difference.

Based on previous findings, we hypothesized that FNDC5 affects the expression of Nrf2 in HCC tissues. After selecting HCC tissues with different levels of expression of FNDC5 *via* immunohistochemical detection, we found that the expression of Nrf2 was also elevated in HCC tissues with high expression of FNDC5 (**Figure 4A**). As the PI3K/Akt pathway regulates the expression of Nrf2, and as we previously found that FNDC5 affects the PI3K/Akt pathway, we evaluated the changes in the expression of PI3K, Akt, and Nrf2 in sorafenib-sensitive HCC, sorafenib-resistant, and *FNDC5*-knockdown resistant cell lines following treatment with the same concentration of sorafenib (**Figure 4B**). Our western blotting results showed that the levels of PI3K, pAkt, and Nrf2 in the nucleus were elevated in the drug-resistant cells compared with sorafenib-sensitive HCC, whereas the activation of the PI3K pathway was diminished and the level of Nrf2 in the nucleus was reduced after the knockdown of FNDC5. To further verify the effect of FNDC5 on the levels of Nrf2 *via* the PI3K/Akt pathway, we treated HCC sorafenib-resistant cells with a PI3K inhibitor for 1 h, and then added sorafenib to detect the levels of Nrf2 and ferroptosis marker proteins. Compared with those in the resistant cells not treated with the PI3K inhibitor, the level of Nrf2 in the nucleus of HCC sorafenib-resistant cells decreased, whereas the level of ferroptosis increased after the administration of the PI3K inhibitor (**Figure 5A**). We further confirmed that the addition of the PI3K inhibitor led to increased levels of ferroptosis in sorafenib-resistant cells as indicated by the levels of ROS and MDA and mitochondrial membrane potential (**Figures 5B** **–D**). Furthermore, we demonstrated that the PI3K inhibitor reversed the resistance of sorafenib-resistant cells as indicated by the flow cytometry detection of apoptotic rate (**Figure 5E**).

### Overexpression of FNDC5 in Hepatocellular Carcinoma Cells Inhibited Sorafenib-Induced Ferroptosis

**Figure 6:**
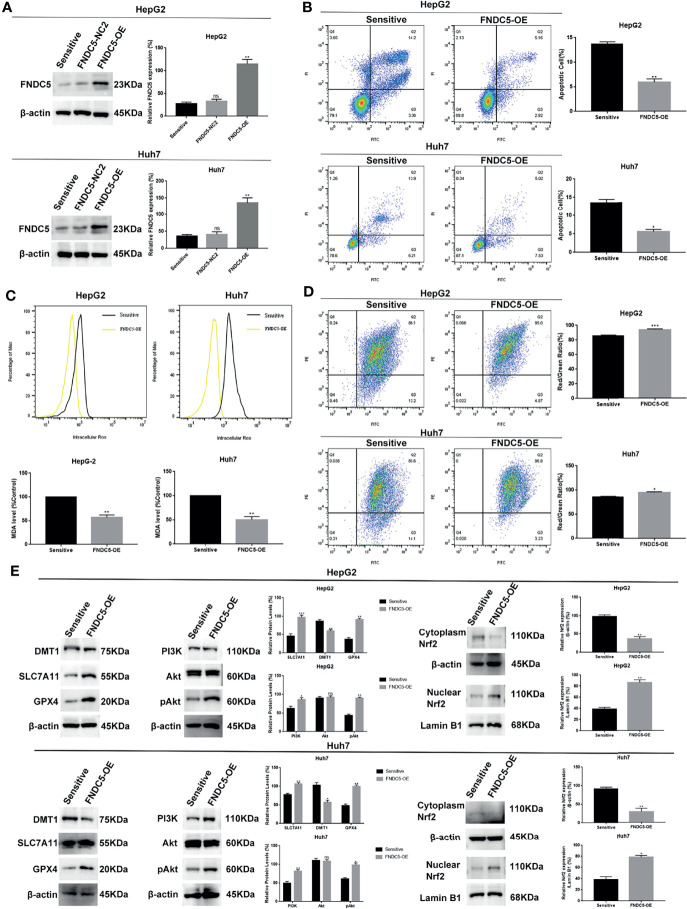
Overexpression of FNDC5 reduces the sensitivity of HCC cells to sorafenib. **(A)** A lentiviral vector was used to overexpress FNDC5; western blotting was performed to verify the overexpression effect. **(B)** After treatment of sorafenib-sensitive HCC and FNDC5-overexpressing HCC cell lines with sorafenib (10 µM) for 24 h, flow cytometry was used to detect the apoptotic rates in both groups. **(C)** ROS and MDA assays were performed to compare the level of ferroptosis between the groups. **(D)** Flow cytometry analysis of alterations in mitochondrial membrane potential in both groups. **(E)** Western blotting for detecting the expression levels of ferroptosis markers and Nrf2-associated pathway proteins in both groups of cells. *Compared with the sorafenib-sensitive HCC cells, *p < 0.05, **p < 0.01, ***p < 0.001. ns, no significant difference.

To further demonstrate that high expression of FNDC5 leads to the resistance of HCC cells to sorafenib, we overexpressed FNDC5 in sorafenib-sensitive HCC cell lines (**Figure 6A**). Annexin-V-FITC/PI staining indicated that the overexpression of FNDC5 in HCC cells led to increased resistance to sorafenib (**Figure 6B**). Furthermore, analyses of the levels of ROS and MDA and mitochondrial membrane potential further confirmed the level of ferroptosis was decreased in sorafenib-sensitive HCC cells with overexpression of FNDC5 compared with sorafenib-sensitive HCC cells (**Figures 6C** **, D**). Finally, western blotting showed that after the overexpression of FNDC5, the PI3K/Akt pathway was activated, the level of Nrf2 in the nucleus was increased, the levels of GPX4 and SLC7721 were increased, whereas the levels of DMT1 and ferroptosis were reduced (**Figure 6E**).

### Alterations in the Level of Expression of FNDC5 Affected the Inhibitory Effect of Sorafenib on Hepatocellular Carcinoma *In Vivo*

**Figure 7:**
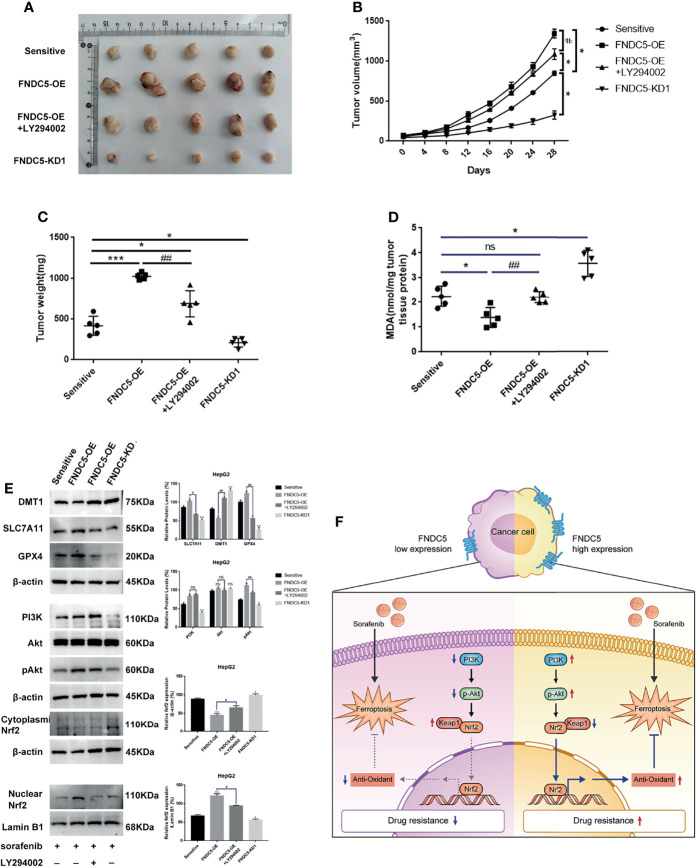
Alterations in the levels of expression of FNDC5 can affect sorafenib-induced ferroptosis in HCC cells *in vivo*. Animals were divided into four groups according to the expression level of FNDC5 and whether or not they were treated with LY294002; all groups were treated with sorafenib. **(A–C)**. Tumor volume in each group was measured every 4 days and final tumor weight was measured. **(D)** The level of MDA in tumor tissues in each group was measured. **(E)** Western blotting for detecting the expression level of ferroptosis markers and Nrf2-associated pathway proteins in tumor tissues in each group. **(F)** Illustration of the mechanistic pathway by which FNDC5 overexpression leads to the resistance of HCC cells to sorafenib. *Compared with the sorafenib-sensitive HCC cells, *p < 0.05, **p < 0.01, ***p < 0.001. #Compared with FNDC5-OE+LY294002, ^#^p < 0.05, ^##^p < 0.01. ns, no significant difference.

We subcutaneously injected HCC cells with different levels of expression of FNDC5 into mice to further investigate whether the changes in the levels of expression of FNDC5 in HCC cells continued to affect the efficacy of sorafenib *in vivo* (**Figures 7A** **–C**). At the same concentration of sorafenib, HCC cells with high expression of FNDC5 exhibited an increased rate of proliferation under the skin of mice and low levels of ferroptosis, whereas *FNDC5*-knockdown HCC cells grew slowly and presented elevated levels of ferroptosis as indicated by western blotting and MDA assays. In addition, we found that treatment with a combination of sorafenib and PI3K inhibitors slowed the growth of HCC cells with high expression of FNDC5 and increased the level of ferroptosis (**Figures 7D** **, E**).

## Discussion

Recent studies have revealed that FNDC5 protects cells from oxidative damage in myocardial inflammation and neurological diseases (20, 21) and reduces the production of ROS, inhibiting the occurrence of ferroptosis in cells (22). As the role of FNDC5 has also been gradually revealed in tumors (23, 24) and in the progression of HCC, FNDC5 has been suggested to play a role in promoting the proliferation of cancer cells. In our previous study, we found that the expression of FNDC5 was higher in cancer than in normal tissues (18, 19). In the present study, we found that the expression of FNDC5 was elevated in sorafenib-resistant cells and that drug-resistant cells with high expression of FNDC5 were not sensitive to treatment with sorafenib, whereas *FNDC5*-knockdown HCC cells were readily killed by sorafenib as observed using the EdU and CCK8 assays. These results suggested that FNDC5 might be one of the factors promoting the development of resistance to sorafenib in HCC.

We also found a substantial decrease in the level of ferroptosis induced by sorafenib in HCC resistant cells with high expression of FNDC5 in *in-vitro* experiments, whereas the level of ferroptosis was elevated in *FNDC5*-knockdown drug-resistant cells. Ferroptosis is a newly identified nonprogrammed apoptotic cell death process (25). It is triggered by the accumulation of iron in cells with decreased levels of glutathione peroxidase (GPX4) (26, 27), increased levels of ROS, and membrane lipid peroxidation reactions that damage cell and mitochondrial membranes (28, 29), leading to cancer cell death (30). Ferroptosis has been shown to promote cancer cell death and exert anticancer effects in a variety of cancers (31). In addition, ferroptosis is an important mode for sorafenib to exert its efficacy during treatment of HCC with sorafenib (6). Using western blotting, we found that after treatment with the same concentration of sorafenib, the levels of xCT and GPX4 were increased, whereas the levels of DMT1 were decreased in drug-resistant HCC cells or *FNDC5*-overexpressing HCC cells compared with those in sorafenib-sensitive HCC cells. However, the opposite results were obtained after the knockdown of FNDC5, indicating that sorafenib-induced ferroptosis was inhibited by the overexpression of FNDC5.

Nrf2 is a major intracellular regulator of oxidative stress, which is generally expressed at low levels in the cytoplasm, and regulated by ubiquitinated degradation mediated by Kelch-like ECH-associated protein 1 (Keap1) (32). When initiating oxidative stress, Nrf2 translocates into the nucleus and binds to the antioxidant response element (ARE), thereby initiating the transcription of antioxidant enzymes (33). Thus, Nrf2 also serves as a factor that inhibits ferroptosis. It has been demonstrated that the activation of Nrf2 reduces the killing effect of sorafenib on HCC cells by inhibiting ferroptosis (34, 35). The PI3K/Akt pathway is an important pathway that regulates the proliferation of tumor cells, and its aberrant activation is one of the mechanisms underlying the progression of many tumors (36–38). Recent studies have shown that Nrf2 is activated by the PI3K/Akt pathway and is involved in regulating tumor growth and the resistance of cancer cells to chemotherapeutic agents (39, 40). In this study, we used immunohistochemistry to investigate whether the expression of Nrf2 was altered in HCC tissues with different levels of expression of FNDC5. Our results showed that Nrf2 was highly expressed in HCC tissues with high expression of FNDC5. Next, we explored the mechanism by which FNDC5 affects the resistance of cells to sorafenib under conditions of co-culturing sorafenib-sensitive HCC, drug-resistant, and FNDC5-knockdown drug-resistant cells with sorafenib. Western blotting showed that, compared with sorafenib-sensitive HCC cells, sorafenib-resistant cells with high expression of FNDC5 presented elevated expression of PI3K, increased levels of downstream pAkt, and elevated levels of Nrf2 in the nucleus. In contrast, in *FNDC5*-knockdown drug-resistant cells, the levels of PI3K and pAkt were reduced, and the level of Nrf2 in the nucleus was also reduced. In resistant cells, we found that PI3K inhibitors decreased the levels of phosphorylated Akt and Nrf2 entering the nucleus but increased the level of ferroptosis. Subsequent validation of the overexpression of FNDC5 further demonstrated that the overexpression of FNDC5 promoted the activation of the PI3K/Akt pathway, increased the level of Nrf2 in the nucleus, and lowered the level of ferroptosis. Finally, *in vivo* experiments also confirmed that FNDC5 inhibited sorafenib-induced ferroptosis by activating the PI3K/Akt/Nrf2 pathway (**Figure 7F**).

## Conclusion

In conclusion, we confirmed that FNDC5 promotes the nuclear translocation of Nrf2 through the activation of the PI3K/Akt pathway, leading to enhanced intracellular antioxidant capacity and ultimately reducing sorafenib-induced ferroptosis. This discovery could contribute to the improvement in the efficacy of sorafenib.

## Data Availability Statement

The original contributions presented in the study are included in the article/supplementary material. Further inquiries can be directed to the corresponding authors.

## Ethics Statement

The studies involving human participants were reviewed and approved by Qingdao Municipal Hospital Ethics Committee. The patients/participants provided their written informed consent to participate in this study. The animal study was reviewed and approved by Qingdao Municipal Hospital Ethics Committee.

## Author Contributions

HL: investigation, formal analysis, and writing – original draft. LZ: methodology and resources. MW: resources and writing – review and editing. KY: resources. ZJ: resources. CZ: writing – review and editing and funding acquisition. GS: conceptualization and project administration, writing review and editing, and funding acquisition. All authors contributed to the article and approved the submitted version.

## Funding

This work was supported by research grants from the National Natural Science Foundation of China (NO. 81601617); Shandong Province Key Research and Development Project (NO. 2018G SF118057).

## Conflict of Interest

The authors declare that the research was conducted in the absence of any commercial or financial relationships that could be construed as a potential conflict of interest.

## Publisher’s Note

All claims expressed in this article are solely those of the authors and do not necessarily represent those of their affiliated organizations, or those of the publisher, the editors and the reviewers. Any product that may be evaluated in this article, or claim that may be made by its manufacturer, is not guaranteed or endorsed by the publisher.
